# Vaccination with Adenovirus Type 5 Vector-Based COVID-19 Vaccine as the Primary Series in Adults: A Randomized, Double-Blind, Placebo-Controlled Phase 1/2 Clinical Trial

**DOI:** 10.3390/vaccines12030292

**Published:** 2024-03-11

**Authors:** Yawen Zhu, Rong Tang, Xiaolong Li, Xiaoqin Chen, Xue Wang, Ying Wang, Ruijie Wang, Fengcai Zhu, Jingxin Li

**Affiliations:** 1 School of Public Health, National Vaccine Innovation Platform, Nanjing Medical University, Nanjing 211166, China; zhuyawen1998zyw@163.com; 2 Jiangsu Provincial Medical Innovation Center, National Health Commission Key Laboratory of Enteric Pathogenic Microbiology, Jiangsu Provincial Center for Disease Control and Prevention, Nanjing 210009, China; tangrongtr@126.com; 3 CanSino Biologics Inc., Tianjin 300457, China; xiaolong.li@cansinotech.com (X.L.); xue.wang@cansinotech.com (X.W.); ying.wang@cansinotech.com (Y.W.); ruijie.wang@cansinotech.com (R.W.); 4 Donghai Center for Disease Control and Prevention, Lianyungang 222300, China; xqchen6800@163.com

**Keywords:** COVID-19 vaccine, aerosolized Ad5-nCoV, safety, immunogenicity, primary series vaccination

## Abstract

This randomized, double-blind, placebo-controlled phase 1/2 trial aimed at evaluating the safety and immunogenicity of Ad5-nCoV via aerosolized or intramuscular or intramuscular–aerosolized routes in SARS-CoV-2-negative adults aged over 18 years. In the phase 1 trial, participants were sequentially enrolled into one of five regimen cohorts: Low-Dose (two doses of aerosolized Ad5-nCoV with 0.5 × 10^10^ viral particles [vps] per dose), Middle-Dose (two doses of aerosolized Ad5-nCoV with 1.0 × 10^10^ vps per dose), High-Dose (two doses of aerosolized Ad5-nCoV with 2.0 × 10^10^ vps per dose), Mixed (intramuscular Ad5-nCoV with 5.0 × 10^10^ vps [first dose] and aerosolized Ad5-nCoV with 2.0 × 10^10^ vps [second dose]), and Single-Dose (one dose of aerosolized Ad5-nCoV with 1.0 × 10^10^ vps). Eligible participants in the phase 2 trial were stratified by 18–59 years old or ≥60 years old and then were sequentially enrolled into one of six regimen cohorts: Low-Dose, Middle-Dose, High-Dose, Mixed, Single-Dose, and Intramuscular (one dose of intramuscular Ad5-nCoV with 1.0 × 10^10^ vps). The intervals between the two doses were 56 days. Participants were randomly allocated in 3:1 (phase 1) and 5:1 (phase 2) ratios to receive either Ad5-nCoV or the placebo in each cohort. This study is registered on ClinicalTrials.gov, NCT04840992. Most adverse reactions that occurred during the solicited period were mild and moderate. One serious adverse event (myelodysplastic syndrome) was considered potentially related to the aerosolized Ad5-nCoV. The GMTs of neutralizing antibodies in the Mixed group were the highest with 57.03 (95% CI: 23.95, 135.80) and 97.37 (95% CI: 74.30, 127.59) in phase 1 and 2 trials, respectively, 28 days after the second dose (*p* < 0.0001), which showed significantly higher immune responses compared to other regimens with aerosolized or intramuscular Ad5-nCoV alone.

## 1. Introduction

The development of effective vaccines and vaccination strategies targeting emerging SARS-CoV-2 variants is crucial for the control of the COVID-19 epidemic. Convidecia, a non-replicating Ad5-vectored COVID-19 vaccine, developed by CanSino Biologics Inc. for intramuscular administration, has demonstrated efficacy against symptomatic or severe COVID-19 in a phase 3 trial [1]. Therefore, Convidecia, along with other viral vector COVID-19 vaccines, including ChAdOx1 nCoV-19 (AZD1222) and Ad26.COV2.S (Johnson & Johnson, New Brunswick, NJ, USA) vaccine, has received recommendation for Emergency Use Listing (EUL) from the World Health Organization (WHO) [2,3,4]. Nevertheless, all these COVID-19 vaccines elicited limited and temporary protection against evolving virus variants, which can evade population immunity [5]. It is widely acknowledged that the route of vaccine administration (intramuscular or aerosol routes) plays a significant role in eliciting immune response in the respiratory mucosa, which could be critical for the vaccine-induced protection against mucosal respiratory viruses like SARS-CoV-2. Thus, a COVID-19 mucosal vaccine that can induce strong mucosal immunity in the respiratory tract is a strategic approach to enhancing the effectiveness of preventing infection and symptomatic diseases and transmission of SARS-CoV-2 [6]. Mucosal vaccines have the added benefit of needle-free administration, which improves accessibility and increases vaccination rates among certain populations [7]. The aerosolized Ad5-vectored COVID-19 vaccine (Ad5-nCoV) through a mucosal immunization routine has shown safety but limited immunogenicity in naïve adults without prior SARS-CoV-2 infection or vaccination history in a pilot study in Wuhan [8]. Therefore, we conducted a randomized, double-blind, placebo-controlled phase 1/2 clinical trial of Ad5-nCoV in order to further explore an optimal vaccine dosage, formulation, and vaccination regimen.

Here, we report the results of the safety and immunogenicity of Ad5-nCoV via aerosolized or intramuscular or intramuscular–aerosolized routes as the primary series vaccination in healthy adults aged 18 years and above in China in 2021.

## 2. Methods

### 2.1. Study Design and Participants

In Donghai County, Jiangsu Province, China, we conducted a randomized, double-blind, placebo-controlled phase 1/2 trial of aerosolized or intramuscular Ad5-nCoV. Healthy adults aged 18 years and above were eligible, without any exposure history to SARS-CoV-2 or who did not receive a COVID-19 vaccine. Individuals who were HIV-positive, who had an axillary temperature > 37.0 °C, or who were seropositive for SARS-CoV-2-specific IgG or IgM were excluded at enrollment. A complete list of inclusion and exclusion criteria is provided in the supplementary data. This study was performed in a dose–escalation manner: from low dose to high dose. The escalation of the dose was paused if any safety risks were noted.

Prior to enrollment, each participant was required to sign written informed consent. The study protocol and informed consent form received approval by the Jiangsu Ethics Committee (JSJK2021-A003-02). This clinical trial was conducted in compliance with the guidelines of Good Clinical Practice of China and the International Conference on Harmonization. This study is registered on ClinicalTrials.gov, NCT04840992.

### 2.2. Randomization and Masking

**Figure 1:**
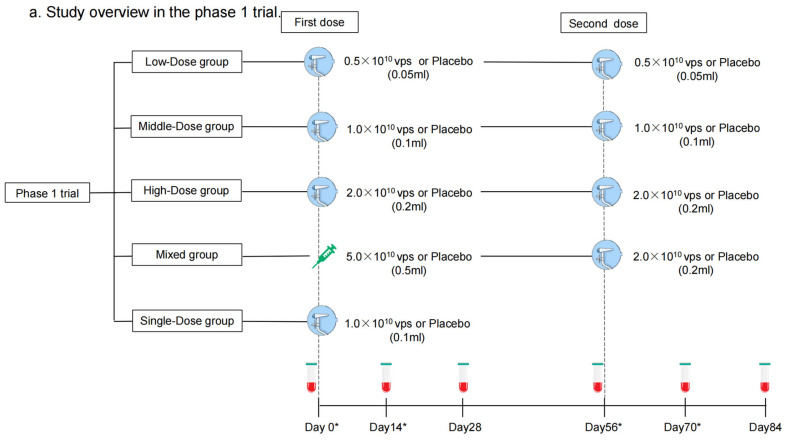

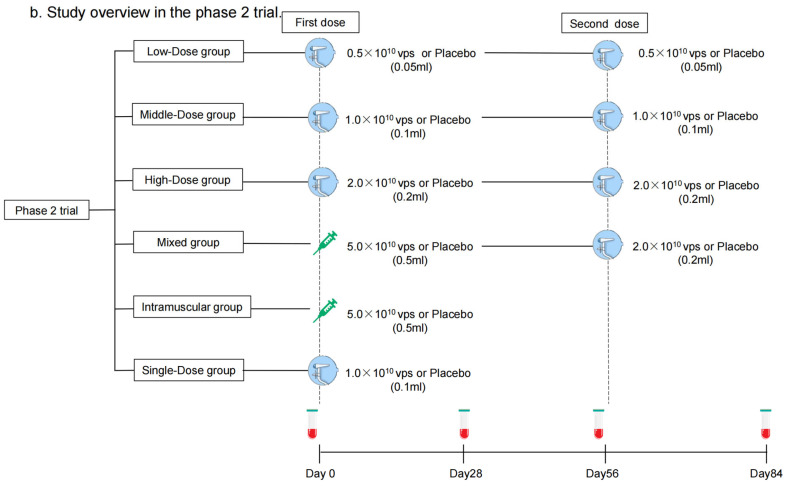
Study overview. Phase 1 trial (**a**) and Phase 2 trial (**b**). Blood samples were collected before vaccination on days 0 and 56. Blood samples were collected for humoral immunity. * means blood samples were collected for cellular immunity on days 0, 14, 56, and 70 as well.

**Figure 2:**
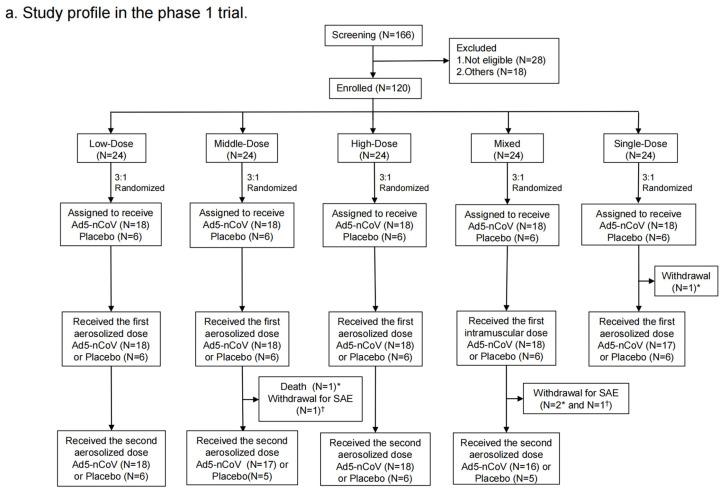

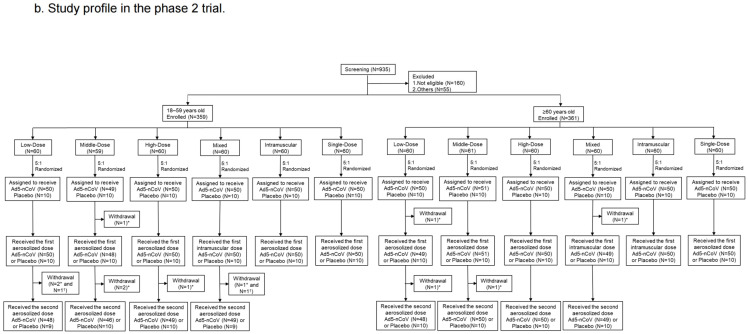
Study profile. Phase 1 trial (**a**) and Phase 2 trial (**b**). * means participants who received Ad5-nCoV; † means participants who received placebo.

Each dose of aerosolized Ad5-nCoV contains replication-defective Ad5 vectors at a concentration of 1.0 × 10^11^ viral particles (vps) per mL. Convidecia, the intramuscular Ad5-nCoV, has been licensed, and was administered intramuscularly in a dose of 0.5 mL. The aerosolized Ad5-nCoV was inhaled at 0.05 mL, 0.1 mL, or 0.2 mL per dose. The placebo only consisted of the vaccine excipients, without any viral particles. In the phase 1 trial, participants were sequentially enrolled into one of five regimen cohorts: Low-Dose (aerosolized Ad5-nCoV with 0.5 × 10^10^ vps per dose, two doses), Middle-Dose (aerosolized Ad5-nCoV with 1.0 × 10^10^ vps per dose, two doses), High-Dose (aerosolized Ad5-nCoV with 2.0 × 10^10^ vps per dose, two doses), Mixed (intramuscular Ad5-nCoV with 5.0 × 10^10^ vp [first dose] and aerosolized Ad5-nCoV with 2.0 × 10^10^ vps [second dose]), and Single-Dose (aerosolized Ad5-nCoV with 1.0 × 10^10^ vps, one dose) groups (Figure 1a). Participants within each cohort were randomly allocated in a 3:1 ratio to receive Ad5-nCoV or a placebo. As to the phase 2 trial, participants were stratified into two age groups: 18–59 years old or ≥60 years old and then were sequentially enrolled into one of six regimen cohorts: Low-Dose, Middle-Dose, High-Dose, Mixed, Single-Dose, and Intramuscular Dose groups (one intramuscular dose of Ad5-nCoV with 1.0 × 10^10^ vps) (Figure 1b). Participants were randomly allocated in a 5:1 ratio to receive either Ad5-nCoV or placebo in each cohort (Figure 2). The interval between the two doses was 56 days. The randomization lists were created by a statistician independently with SAS (version 9.4). The assignment of the specific regimen cohorts was available for both participants and investigators, but they were masked for the treatment allocation within each regimen. The enrollment of the latter cohort was initiated after all the participants in the previous cohort had finished a 14-day safety observation.

### 2.3. Procedures

All the participants were observed for reactions related to vaccines for 30 min after each dose at the vaccination sites. They were guided to record local or systemic adverse events on paper diary cards for the first 14 days after each vaccination. From days 15 to 28 after each dose, safety information was gathered through spontaneous reports from participants as well as regular visits by the investigators. Serious adverse events (SAEs) were documented throughout the trial and were collected for up to 12 months after the whole immunization. Investigators assessed the severity grade of the adverse events according to the guidelines released by the China State Food and Drug Administration [9]. Meanwhile, the investigators made a decision of whether adverse events and vaccination were causal associated.

Participants donated blood samples on day 0 immediately before vaccination and on days 14 and 28 post-vaccination for the detection of specific antibody responses against the receptor-binding domain (RBD) using ELISA kit (Vazyme Medical Technology, Nanjing, China) [10]. Indirect ELISA assays were performed to measure RBD-specific IgG and sera IgA responses with the lowest limit titer of 1:10. The assessment of neutralizing antibody (NAb) responses to live SARS-CoV-2 virus was determined by a 50% plaque reduction neutralization assay [10] with diluting serum sample starting at a 1:4 ratio and then performing a two-fold gradient dilution. A serum titer ≥ 1:4 for SARS-CoV-2 live virus NAb was defined as seropositivity. Seroconversion was considered as at least a four-fold increase in the antibody titer post-vaccination. For undetectable antibody titers in serum, values equivalent to half the detection limits were assigned for calculation. Additionally, peripheral blood mononuclear cells (PBMCs) were isolated from whole blood on days 0, 14, 15, and 70 in phase 1 trial. Enzyme-linked immunospot (ELISpot) assay (Mabtech, Stockholm, Sweden) was utilized to quantify specific T-cell responses of cytokine secretion from interferon-γ (IFN-γ), tumor necrosis factor-α (TNF-α), and interleukin (IL-4, 5, and 13). The results are expressed as the number of positive spot counts per 10^5^ cells, as reported previously [10]. Positive ELISpot response was defined as the difference between the mean of peptide wells and negative control wells greater than 5 and the ratio greater than 2.1. The pre- and post-vaccination anti-Ad5 neutralizing antibody titers were detected using a serum neutralization assay [11]. 

### 2.4. Outcomes

The primary endpoint for safety in both phase 1 and 2 trials was the occurrence rates of adverse reactions occurring within 14 days after each vaccination and that of SAEs during the 12-month follow-up period. Additionally, the primary immunogenicity endpoint was the geometric mean titer (GMT) of neutralizing antibody responses against SARS-CoV-2 live virus on day 28 day after the last vaccination in the phase 2 trial.

The secondary safety outcomes included the incidence of adverse reactions/events occurring within 30 min, 7 days, and 28 days after each dose. The secondary immunogenicity outcomes comprised GMT, seroconversion rate (SR), and geometric mean fold increase (GMFI) for RBD-specific IgG and sera IgA antibody responses at day 0 before each dose, day 28 after the first dose, and days 14 and 28 after the second dose. Seroconversion rate and GMFI in neutralizing antibody responses against SARS-CoV-2 live virus 28 days after the last dose were also secondary endpoints. Furthermore, the cellular immunogenicity endpoints were levels of five cytokine-producing cells at days 0 and 14 after each dose in the phase 1 trial.

### 2.5. Statistical Analysis

In accordance with the Technical Guidelines for Vaccine Clinical Trials, phase 1 clinical trials are usually pilot scale studies with 20–30 participants in each group. According to the results of Intramuscular Ad5-nCoV in phase 2 clinical trial before, the cohort of participants in the Intramuscular group is considered as the positive control group, assuming that the variation in GMT caused by different batches of vaccines and non-simultaneous testing is similar, with a standard deviation of 0.6, at a significance level of α = 0.025, ensuring an 80% power, and setting the non-inferiority margin δ at −0.3. The number of participants receiving the experimental vaccine in each group should be approximately 64, matching the number in the Intramuscular group in a 1:1 ratio. Considering the dropout rate and stratification, it was determined that 100 participants receiving the experimental vaccine were needed in each group. The age groups were divided into 18–59 years and 60 years and above, with a 5:1 allocation ratio within each group for the experimental vaccine or placebo, so that the total sample size was 720 cases.

We utilized the Chi-square test or Fisher’s exact test when necessary for analyzing categorical outcomes, for both safety and immunogenicity. The 95% confidence intervals (CIs) for all categorical outcomes were calculated using the Clopper–Pearson method. GMTs and corresponding 95% CIs were calculated when obeying standard normal distribution of the log-transformed antibody titer. The ANOVA method was employed to compare the log-transformed antibody titer. In cases where significant differences were observed among all groups, pairwise comparisons were conducted. Two-sided *p* values < 0.05 were considered to be significant. All the statistical analyses were performed using SAS software (version 9.4).

## 3. Results

### 3.1. Study Participants

From 20 April to 24 May 2021, a total of 166 participants were recruited. Of them, 28 were excluded for being not eligible and 18 of them were excluded for other reasons. Finally, 120 individuals were enrolled and randomized into the following groups in the phase 1 trial: 18 participants in the Low-Dose group, 18 in the Middle-Dose group, 18 in the High-Dose group, 18 in the Mixed group, 18 in the Single-Dose group, and 30 in the placebo group (Figure 2a). Demographic characteristics of the participants at baseline were comparable among groups (Table 1). There were 42 (35.3%) participants with high pre-existinganti-Ad5 antibodies, while there were 77 (64.7%) with low or negative pre-existing immunity. A total of 119 participants were vaccinated with at least one dose, of which 93 participants received the second dose.

From 10 May to 11 June 2021, 935 participants were recruited but 160 of them were not eligible and 55 of them were excluded for other reasons in the phase 2 trial. At last, a total of 359 participants aged 18–59 years old and 361 participants aged ≥ 60 years old (60–88-year-olds) were enrolled and randomly assigned to the Low-Dose, Middle-Dose, High-Dose, Mixed, Intramuscular, Single-Dose and placebo groups (Figure 2b). The baseline characteristics of the participants were balanced among the groups. A total of 228 (31.8%) had high pre-existing anti-Ad5 antibodies, and 489 (68.2%) had low or negative pre-existing immunity in the phase 2 trial (Table 1). 

### 3.2. Safety

**Figure 3:**
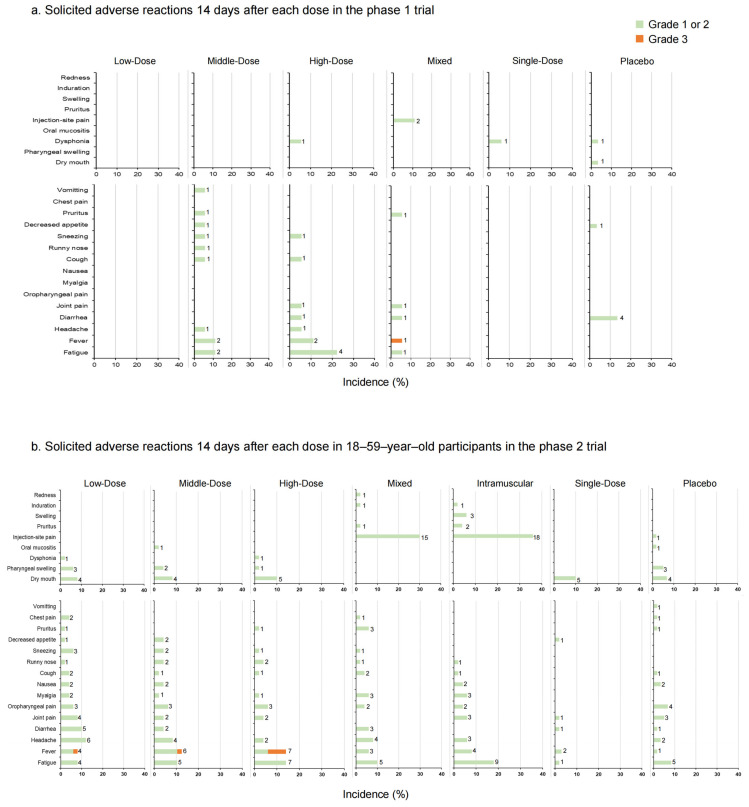

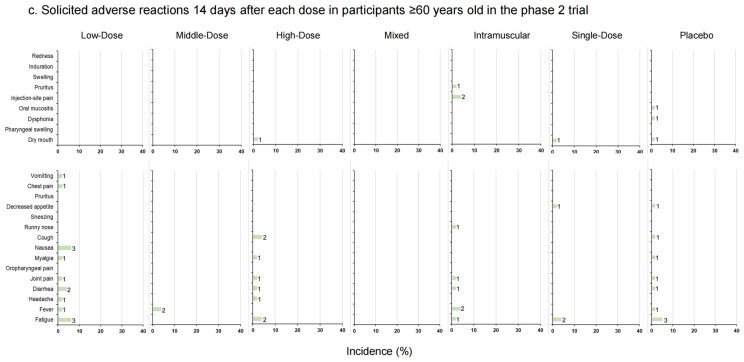
The incidence rates of solicited local and systemic adverse reactions 14 days after each vaccination in phase 1 trial (**a**); solicited local and systemic adverse reactions 14 days after each dose in 18–59–year–old participants in phase 2 trial (**b**); solicited local and systemic adverse reactions 14 days after each dose in participants ≥ 60 years old in phase 2 trial (**c**). The data on the right of the bars indicate the number of participants who reported adverse reactions.

In the phase 1 trial, adverse reactions occurred in two (11.11%) participants in the Low-Dose group, four (22.22%) in the Middle-Dose group, six (33.33%) in the High-Dose group, five (27.78%) in the Mixed group, and one (5.88%) in the Single-Dose group 14 days post-vaccination (Figure 3a, Table S2). In the placebo group, five (16.67%) participants reported adverse reactions. No solicited adverse reactions were observed in the Low-Dose group. The occurrence rate of adverse reactions increased with the higher doses administered. Dysphonia was the most common local symptom in participants who received aerosolized Ad5-nCoV (5.56% in the High-Dose group, 5.88% in the Single-Dose group, and 3.33% in the placebo group). Injection-site pain (11% in the Mixed group) was the most common local symptom in participants who received injection administration. The most common systemic symptom was fatigue, with the highest occurrence rate in the High-Dose group (22.22%) and the next-highest rates were in the Middle-Dose group (11.11%) and the Mixed group (5.56%) (*p* = 0.013). The majority of adverse reactions were of mild or moderate severity, with only one Grade 3 adverse reaction (fever) monitored in the Mixed group. During the study period, 15 serious adverse events were documented by six participants. One 64-year-old female participant in the High-Dose group reported myelodysplastic syndrome approximately 7 months after the second vaccination, which was potentially related to the aerosolized Ad5-nCoV and resulted in hospitalization (Table S1). 

In the phase 2 trial, 18 participants (36.00%) versus 7 participants (14.29%) in the Low-Dose group (18–59 years old versus ≥ 60 years old), 15 participants (31.25%) versus 2 participants (3.92%) in the Middle-Dose group, 12 participants (24.00%) versus 3 participants (6.00%) in the High-Dose group, 23 participants (46.00%) versus 1 participant (2.04%) in the Mixed group, 25 participants (50.00%) versus 7 participants (14.00%) in the Intramuscular group, 9 participants (18.00%) versus 2 participants (4.00%) in the Single-Dose group and 14 participants (23.33%) versus 5 participants (8.33%) in the placebo group (*p* = 0.003 versus *p* = 0.108) reported at least one adverse reaction (Figure 3b,c, Table S3). In participants aged 18–59, the incidence rates of adverse reactions in the Mixed and Intramuscular groups were higher than in other groups within 14 days after vaccination. Dry mouth was the most frequent local adverse reaction via inhalation administration, and the most frequent local adverse reaction via injection was pain. Meanwhile, in the systemic adverse reactions, fever was the most common one. No significant difference between the occurrence rates of total adverse reactions was observed in participants aged 60 and over (*p* = 0.108). Most of the adverse reactions were generally mild or moderate in severity and resolved within several days after onset, while six adverse reactions graded as 3, fever, were documented by one (2.00%) participant in the Low-Dose group, one (2.08%) participant in the Middle-Dose group, and four (8.00%) participants in the High-Dose group of 18–59-year-olds. There was a tendency that severe fever was more likely to occur with aerosolized administration. Nineteen participants reported serious adverse events after vaccination, but none of them were related to trial vaccines (Table S1).

### 3.3. Immunogenicity

**Figure 4:**
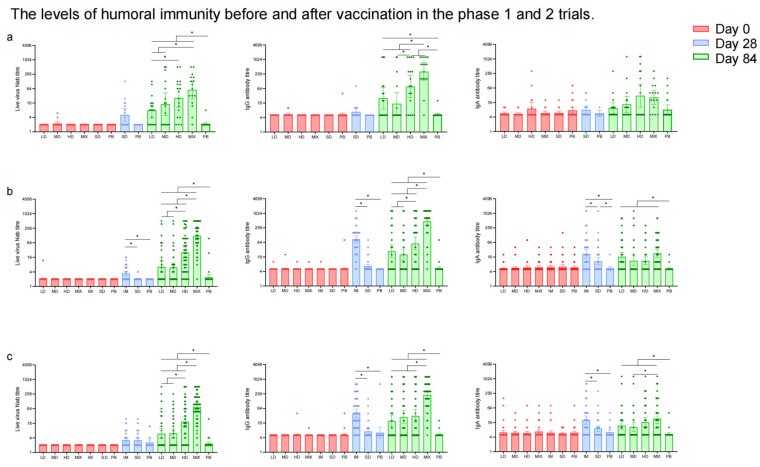
Showing GMTs of neutralizing antibodies to live SARS-CoV-2, RBD-IgG antibodies, and sera IgA antibodies in phase 1 trial (**a**); GMTs of neutralizing antibodies to live SARS-CoV-2, RBD-IgG antibodies, and sera IgA antibodies in 18–59-year-old populations in phase 2 trial (**b**); GMTs of neutralizing antibodies to live SARS-CoV-2, RBD-IgG antibodies, and sera IgA antibodies in ≥60-year-old populations in phase 2 trial (**c**) before vaccination and 28 days after the last vaccination. Error bars indicate 95% CIs. * means *p* < 0.05 in pairwise comparison.

**Figure 5:**
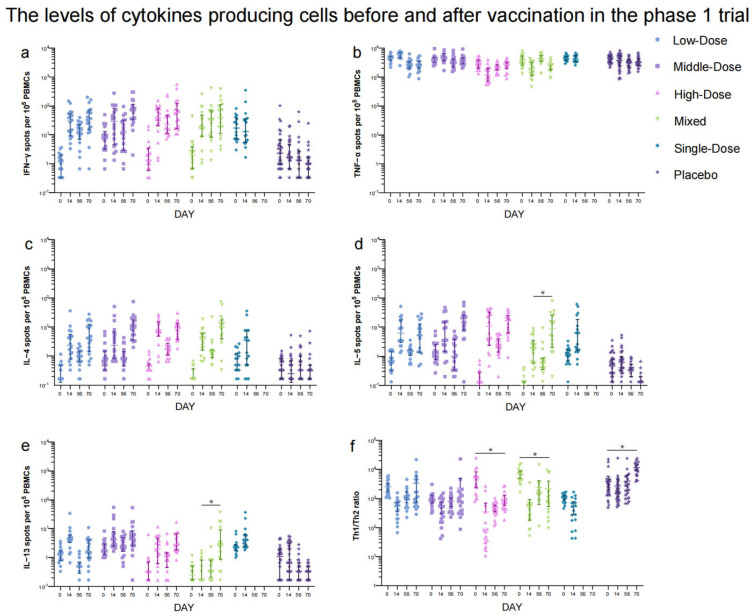
IFN−γ (**a**), TNF−α (**b**), IL−4 (**c**), IL−5 (**d**), IL−13 (**e**) cytokine-producing cell responses and Th1/Th2 (**f**). Data are mean (95% CI) of positive spot counts per 10^5^ PBMCs. Error bars indicate 95% CIs. * means *p* < 0.05.

In the phase 1 trial, NAb responses to live SARS-CoV-2 were significantly higher in the Low-Dose (GMT: 7.93 [95% CI: 4.14, 15.19]), Middle-Dose (GMT: 14.36 [95% CI: 4.84, 42.61]), High-Dose (GMT: 26.32 [95% CI: 10.57, 65.54]), and Mixed (GMT: 57.03 [95% CI: 23.95, 135.80]) groups compared to the placebo group (GMT: 2.12 [95% CI: 1.88, 2.41]) 28 days after the second vaccination (Figure 4a, Table S5). Among these regimen groups, the Mixed group showed the highest neutralizing antibody titers (*p* < 0.0001). Similarly, the GMTs of IgG antibodies were higher in the Low-Dose, Middle-Dose, High-Dose, and Mixed groups compared to the placebo group 28 days after the second dose of which the Mixed group (320.00 [95% CI: 136.37, 750.92]) induced the highest IgG antibody titers (*p* < 0.0001) (Table S6). The GMTs of IgA antibodies in the High-Dose (30.55 [95% CI: 10.07, 92.69]) and Mixed groups (25.94 [95% CI: 13.06, 51.51]) were higher than those in the Low-Dose (9.26 [95% CI: 5.47, 15.69]), Middle-Dose (13.30 [95% CI: 6.12, 28.94]), and placebo (8.10 [95% CI: 5.23, 12.54]) groups (*p* < 0.0001) 28 days after the second dose (Table S7). The difference in NAb, IgG, and sera IgA antibody levels between the Single-Dose and placebo groups was not statistically significant. The seroconversion rates and GMFIs of neutralizing antibodies, IgG, and IgA showed a similar trend to the GMTs among the Low-Dose, Middle-Dose, High-Dose, Mixed, and placebo groups after the second vaccination (Tables S5–S7). The IFN-γ responses (39.59 [95% CI: 19.55, 59.64], 50.96 [95% CI: 17.35, 84.58], 52.37 [95% CI: 31.62, 73.12], 41.57 [95% CI: 11.54, 71.61], and 45.31 [95% CI: 1.35, 89.28] spots/10^5^ PBMCs) were higher in all the regimen groups compared to the placebo group (3.62 [95% CI: 1.75, 5.50] spots/10^5^ PBMCs) after the first dose (Figure 5), but there was no significant increase in IFN-γ responses between the two doses. Additionally, no significant difference was observed in TNF-α- and IL-4-producing cells before and after vaccination. The levels of IL-5 responses (81.42 [95% CI: 26.76, 136.07] versus 12.81 [95% CI: 6.65, 18.98] spots/10^5^ PBMCs) and IL-13 responses (0.89 [95% CI: 0.40, 1.37] versus 13.04 [95% CI: 2.53, 23.55] spots/10^5^ PBMCs) in the Mixed group increased significantly 14 days following the second dose compared to the first vaccination.

NAbs to SARS-CoV-2 live virus were comparable among groups at baseline in the phase 2 trial (Figure 4b,c, Table S8). The GMT of neutralizing antibodies in the Intramuscular group was higher than in the Single-Dose and placebo groups (*p* < 0.0001) in participants aged 18–59 years, while there was no significant difference in the GMTs of neutralizing antibodies among the Intramuscular, Single-Dose, and placebo groups in participants aged over 60 after the first vaccination. The GMTs of neutralizing antibodies to the SARS-CoV-2 live virus in the Mixed group (124.97 [95% CI: 85.45, 182.76] and 76.25 [95% CI: 51.88, 112.07]) were the significantly highest compared to those in the Low-Dose, Middle-Dose, and High-Dose groups 28 days after the second dose (*p* < 0.0001), and the above four regimen groups were higher than those in the placebo groups (*p* < 0.0001) in both age groups. The seroconversion rates and GMFIs of NAb in the Mixed group were the highest, followed by the High-Dose group, and those in the placebo group were the lowest (*p* < 0.0001) (Table S8). The results in participants with low (titer ≤ 1:200) or high (titer > 1:200) pre-existing anti-Ad5 antibodies indicated that high pre-existing anti-Ad5 immunity would bring down the neutralizing antibody responses elicited by Ad5-nCoV to a certain degree (Figure S1).

In the phase 2 trial, RBD-IgG antibodies and sera IgA were comparable across groups before vaccination (Tables S9 and S10). The GMTs of IgG and sera IgA antibodies in the Intramuscular group were higher than in the Single-Dose group and the placebo group (*p* < 0.0001) after the first vaccination in both age groups. The GMTs of IgG antibodies in the Mixed group (472.58 [95% CI: 320.91, 695.94] in 18–59-year-old participants and 227.88 [95% CI: 152.36, 340.83] in ≥60-year-old participants) were higher than the Low-Dose, Middle-Dose, and High-Dose groups, and those in the placebo groups were the lowest after the second vaccination (*p* < 0.0001). Following the second dose, the GMTs of IgA antibodies in the four regimen groups were higher than that in the placebo group (*p* < 0.0001), but no significant difference among the four groups above was observed (Figure 4b,c, Tables S9 and S10). The seroconversion rates and GMFIs of IgG and IgA showed a similar trend to the GMTs among the Low-Dose, Middle-Dose, High-Dose, Mixed, and placebo groups after the second vaccination.

## 4. Discussion

Our data indicate that the safety profile of two doses of aerosol vaccination in adults aged 18 years and older differed from vaccination with two intramuscular injections [8], because the incidence rate of adverse events of the former was lower than the latter. The incidence of adverse reactions via aerosol vaccination tended to increase with higher doses. The adverse reaction incidence rate of participants aged 18–59 years was higher than that of participants aged ≥ 60 years. The incidence of adverse reactions resulting from two doses of aerosolized Ad5-nCoV 56 days apart was lower than a previous study with a 28-day interval in Wuhan [8]. Systemic adverse reactions were more likely to occur with aerosolized inhalation compared to intramuscular injection. While one case of myelodysplastic syndrome occurred, associated with thrombocytopenia, it was rare overall and has also been reported with other adenoviral vector vaccines [12]. The very low incidence of pain in the participants ≥ 60 years of age may be attributable to the fact that old people generally have a higher pain threshold. There was one death in the phase 1 trial (car accident) and three deaths (two for acute myocardial infarction and one for cerebral hemorrhage) in the phase 2 trial that were considered to be irrelevant to the study vaccines by the investigators. In general, the aerosolized Ad5-nCoV vaccine demonstrates a favorable safety profile, especially in the elderly population, which is in line with previous studies [8].

The data presented in our study declare that a second dose of the Ad5-nCoV through inhalation after an initial dose of the Ad5-nCoV administered via injection, gave rise to significantly higher levels of humoral immunity compared to two doses of inhalation alone or a single dose of intramuscular vaccination. This was evident through increased levels of RBD-binding IgG antibodies and neutralizing antibodies. Additionally, there appears to be a strong correlation between the titers of RBD-binding IgG antibodies and neutralizing antibodies (r = 0.8519, 0.8023, and 0.7816, Figure S2). Although RBD-binding IgG titers with two aerosolized doses were lower than those with one intramuscular injection, neutralizing antibody titers in the aerosol vaccination groups were higher than those with intramuscular injection. This finding indicates that the main antibody compositions produced by various vaccination routes differ, with aerosol vaccination potentially eliciting a greater percentage of neutralizing antibodies and a lower proportion of IgG to total antibodies, compared with intramuscular vaccination. Furthermore, the study observed a significant dose–response relationship with different doses of orally aerosolized Ad5-nCoV. The administration of 2.0 × 10^10^ viral particles induced higher levels of humoral immunity compared to lower doses. However, no substantial impact on cellular immunity was observed with the mixed vaccination approach employed in this trial. The ratio of Th1 and Th2 tended to decrease 14 days after vaccination, suggesting that aerosolized Ad5-nCoV in our study may induce Th2-biased cellular immunity (Figure 5f). This finding was in line with the increase in IL-5- and IL-13-producing cells. Moreover, the antibodies were positively related to the levels of IL-4-, IL-5-, and IL-13-producing cytokines (*p* < 0.0001), which represented Th2-polarized cells (Figure S3c–e). Th2 cytokines were known to stimulate the production of antibodies [13]. As to the IFN-γ and TNF-α cytokines (Figure S3a,b), there was no or negative correlation with antibodies. Currently, the aerosolized Ad5-nCoV is the first orally aerosolized SARS-CoV-2 vaccine to be granted for emergency use as a booster dose following inactivated SARS-CoV-2 vaccines for initial immunization in China [14]. A heterologous dose of aerosolized Ad5-nCoV as a booster has been demonstrated to be safe and to augment effective immune responses in both healthy adults immunized with CoronaVac or Covilo for two or three doses [10,15]. These findings highlight the potential of aerosolized Ad5-nCoV as a booster strategy to enhance immune responses against COVID-19 [16,17]. However, the studies on aerosolized Ad5-nCoV as primary immunization were limited. Our data confirm that two doses of Ad5-nCoV were safe and induced effective antibody responses in adults without prior COVID-19 vaccination history, which provided further support for aerosolized Ad5-nCoV as the primary series vaccination.

There are several limitations in our study. First of all, we did not evaluate the antibody levels against other SARS-CoV-2 variants. Secondly, long-term immunogenicity and safety were not evaluated. Thirdly, the specific secretory IgA concentration in the saliva or cytokines more related to mucosal responses like Th17 were not assessed after vaccination. An additional limitation of the study is that the vaccine was tested in naïve participants, while a large part of the population has now been exposed to SARS-CoV-2 or vaccinated with a COVID-19 vaccine.

## 5. Conclusions

In conclusion, the aerosolized Ad5-nCoV demonstrates a favorable safety profile, but it is limited in the immunogenicity of two doses of aerosolized Ad5-nCoV alone. The administration of an aerosolized Ad5-nCoV vaccine 56 days after the initial intramuscular injection of Ad5-nCoV brings about robust IgG and neutralizing antibody responses, serving as an effective primary series vaccination. Further studies are required to assess the long-term safety and immunogenicity of aerosolized Ad5-nCoV. The viral vector platform is meaningful to be utilized to develop mucosal vaccines against other challenging respiratory infectious diseases.

## Figures and Tables

**Table 1:** Baseline demographic characteristics in the phase 1 and 2 trials.

Phase 1	Low-Dose	Middle-Dose	High-Dose	Mixed	Single-Dose	Placebo	
N	18	18	18	18	17	30	
Age (SD), years	45.4 (17.4)	51.1 (14.4)	50.9 (15.5)	57.4 (14.2)	50.3 (15.5)	56.5 (13.9)	
18–59	12 (66.67)	13 (72.22)	12 (66.67)	11 (61.11)	10 (58.82)	14 (46.67)	
≥60	6 (33.33)	5 (27.78)	6 (33.33)	7 (38.89)	7 (41.18)	16 (53.33)	
Sex (%)							
Male	9 (50.0)	4 (22.2)	7 (38.9)	12 (66.7)	5 (29.4)	17 (56.7)	
Female	9 (50.0)	14 (77.8)	11 (61.1)	6 (33.3)	12 (70.6)	13 (43.3)	
Body-mass index (SD), kg/m^2^	25.4 (3.8)	25.0 (2.5)	25.3 (3.9)	25.5 (2.6)	26.3 (3.4)	24.9 (3.5)	
Pre-existing adenovirus type 5 neutralizing antibody							
≤1:200, titer	5 (27.8)	6 (33.3)	7 (38.9)	6 (33.3)	6 (35.3)	12 (40.0)	
>1:200, titer	13 (72.2)	12 (66.7)	11 (61.1)	12 (66.7)	11 (64.7)	18 (60.0)	
**Phase 2**	**Low-Dose**	**Middle-Dose**	**High-Dose**	**Mixed**	**Intramuscular**	**Single-Dose**	**Placebo**
N	99	99	100	99	100	99	120
Age (SD), years	56.8 (12.9)	57.5 (12.3)	58.8 (13.4)	56.7 (14.0)	58.9 (12.7)	57.7 (11.8)	55.8 (13.8)
18–59	50 (50.5)	48 (48.5)	50 (50.0)	50 (50.5)	50 (50.0)	50 (50.5)	60 (50.0)
≥60	49 (49.5)	51 (51.5)	50 (50.0)	49 (49.5)	50 (50.0)	49 (49.5)	60 (50.0)
Sex							
Male	42 (42.4)	50 (50.5)	56 (56.0)	54 (54.5)	45 (45.0)	48 (48.0)	50 (41.7)
Female	57 (57.6)	49 (49.5)	44 (44.0)	45 (45.5)	55 (55.0)	52 (52.0)	70 (58.3)
Body-mass index (SD), kg/m^2^	26.7 (3.2)	25.7 (3.2)	26.0 (3.8)	25.3 (3.6)	25.5 (4.2)	25.8 (3.7)	25.4 (3.5)
Pre-existing adenovirus type 5 neutralizing antibody							
≤1:200, titer	29 (29.3)	24 (24.2)	37 (37.0)	37 (37.4)	27 (27.0)	38 (38.4)	36 (30.0)
>1:200, titer	70 (70.7)	75 (75.8)	63 (63.0)	62 (62.6)	73 (73.0)	62 (61.6)	84 (70.0)

## Data Availability

The data presented in this study are available upon request from the corresponding author.

## Supplementary Materials

The following supporting information can be downloaded at https://www.mdpi.com/article/10.3390/vaccines12030292/s1, Figure S1: Live virus Nab titer in participants with low or high pre-existing anti-Ad5 antibodies. Figure S2: The correlation between neutralizing antibodies and IgG antibodies. Figure S3: The correlation between different cytokine spots and antibodies after vaccination in phase 1 trial; Table S1: Severe adverse events after vaccination.; Table S2: Adverse reactions within 14 days after vaccination in phase 1 trial; Table S3: Adverse reactions within 14 days after vaccination in phase 2 trial; Table S4: Unsolicited adverse reactions in phase 2 trial; Table S5: GMT, GMFI, and seroconversion rates of neutralizing antibodies to live SARS-CoV-2 in the phase 1 trial; Table S6: GMT, GMFI, and seroconversion rates of SARS-CoV-2 RBD-specific IgG in the phase 1 trial; Table S7: GMT, GMFI, and seroconversion rates of sera IgA in the phase 1 trial; Table S8: GMT, GMFI, and seroconversion rates of neutralizing antibodies to live SARS-CoV-2 in the phase 2 trial; Table S9: GMT, GMFI, and seroconversion rates of SARS-CoV-2 RBD-specific IgG in the phase 2 trial; Table S10: GMT, GMFIs and seroconversion rates of sera IgA in the phase 2 trial.
