# Supplementary material for: Vaccination with Adenovirus Type 5 Vector-Based COVID-19 Vaccine as the Primary Series in Adults: A Randomized, Double-Blind, Placebo-Controlled Phase 1/2 Clinical Trial
Source: Vaccines (Basel). 2024 Mar 11;12(3):292. doi: 10.3390/vaccines12030292 (PMC10975308; doi:10.3390/vaccines12030292)
Supplement: Supplementary file 1 [file vaccines-12-00292-s001.zip › vaccines-2873197-supplementary.pdf]

Supplementary data

Inclusion and exclusion criteria:

Inclusion criteria:

- ✓ Aged 18 and over;
- ✓ Obtain informed consent from the subjects themselves and sign the informed consent form;
- ✓ the subjects are able and willing to comply with the requirements of the clinical trial protocol and complete follow-up;
- ✓ HIV-negative;
- ✓ Without nasal or oral diseases, such as acute rhinitis (sinusitis), allergic rhinitis, oral ulcers, pharyngeal redness, etc.;
- ✓ SARS-CoV-2 specific IgG or IgM negative in serum;
- ✓ Axillary temperature≤37.0°C;
- ✓ Without any exposure history to SARS-CoV-2 before.

Exclusion criteria:

Exclusion for the first dose:

- ✓ With severe cardiovascular diseases such as arrhythmia, conduction block, myocardial infarction, severe uncontrolled hypertension (on-site measurement: systolic blood pressure ≥160mmHg, diastolic blood pressure ≥100mmHg), etc.
- ✓ Abnormal laboratory test results deemed clinically significant (including white blood cell count, lymphocyte count, eosinophil count, neutrophil count, platelet count, hemoglobin level, alanine aminotransferase (ALT), aspartate aminotransferase (AST), total bilirubin, fasting blood glucose, creatinine, activated partial thromboplastin time). (Only for Phase I volunteers)
- ✓ Respiratory rate ≥17 (breaths/minute).
- ✓ With lung function abnormalities such as asthma, chronic obstructive pulmonary disease (COPD), pulmonary fibrosis, etc.
- ✓ Vaccinated against COVID-19.
- ✓ With symptoms of upper respiratory tract infection.
- ✓ With a history or family history of seizures, epilepsy, brain disorders, or mental illnesses.
- ✓ Allergic to any component of the study vaccine, with a history of severe vaccine allergic reactions, allergies, or autoimmune diseases.
- ✓ With acute febrile illnesses or infectious diseases.
- ✓ With a history of SARS.
- ✓ With severe chronic diseases or conditions that are not well-controlled, such as diabetes, thyroid disorders, etc.
- ✓ With congenital or acquired angioedema/neurogenic edema.
- ✓ With urticaria (hives) in the year prior to receiving the experimental vaccine.
- ✓ Without a spleen or with non-functioning spleen.
- ✓ With thrombocytopenia or other clotting disorders (may contraindicate intramuscular injection).
- ✓ Experiencing fainting during intramuscular injections.
- ✓ Received immunosuppressive therapy (oral or intravenous for more than 14 days), anti-allergy treatment, cytotoxic therapy, or inhaled corticosteroids for the past 6 months.
- ✓ Received blood products within 4 months prior to receiving the experimental

vaccine.

✓ Received other investigational drugs within 1 month prior to receiving the experimental vaccine.

✓ Received attenuated live vaccines within 1 month prior to receiving the experimental vaccine.

✓ Received subunit or inactivated vaccines within 14 days prior to receiving the experimental vaccine.

✓ Receiving anti-tuberculosis treatment or in the active phase of tuberculosis.

✓ Females with a positive pregnancy test, pregnant or breastfeeding women, or women planning to become pregnant during the entire study period. (Pregnancy tests are only conducted in female volunteers of childbearing potential)

✓ Individuals who, in the opinion of the investigator, are not suitable for the study protocol due to various medical, psychological, social, or other conditions that contradict the study protocol or may affect the participant's ability to provide informed consent.

Exclusion criteria for the second dose:

✓ With a severe allergic reaction after the previous dose of the vaccine.

✓ With a severe adverse reaction causally related to the previous dose of the vaccine.

✓ Individuals who develop new conditions or meet exclusion criteria that were not present or met during the selection for the first dose, as determined by the investigator.

✓ Other exclusion criteria as determined by the investigator.

Figure S1. Live virus Nab titer in participants with different pre-existing anti-Ad5 immunity.

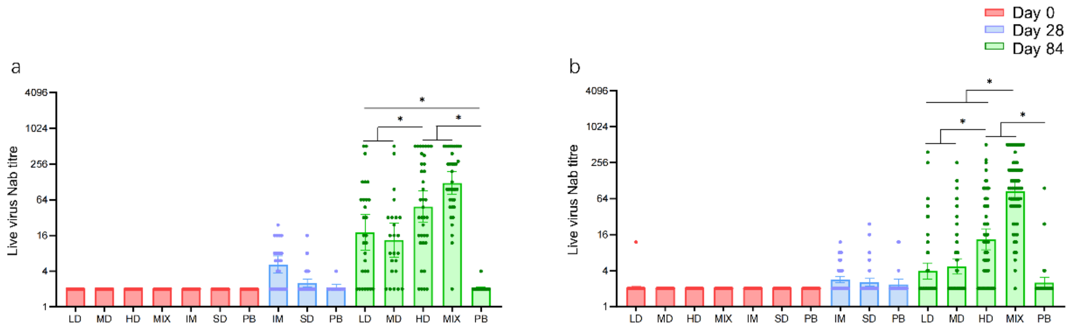

Live virus Nab titer in participants with low (titer ≤1:200) pre-existing anti-Ad5 antibodies (a) and participants with high (titer >1:200) pre-existing anti-Ad5 antibodies (b).

Figure S2. The correlation between neutralizing antibodies and IgG

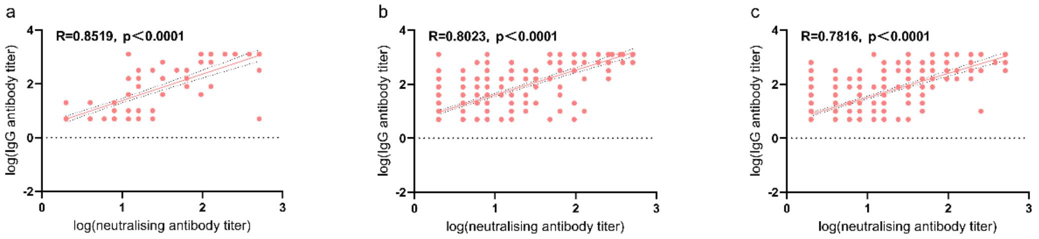

The correlation between neutralizing antibodies and IgG in phase 1 trial (a); in 18-59-year-old participants in phase 2 trial (b); in ≥ 60-year-old participants in phase 2 trial (c).

Figure S3. The correlation between different cytokines spots and antibodies after vaccination in phase 1 trial

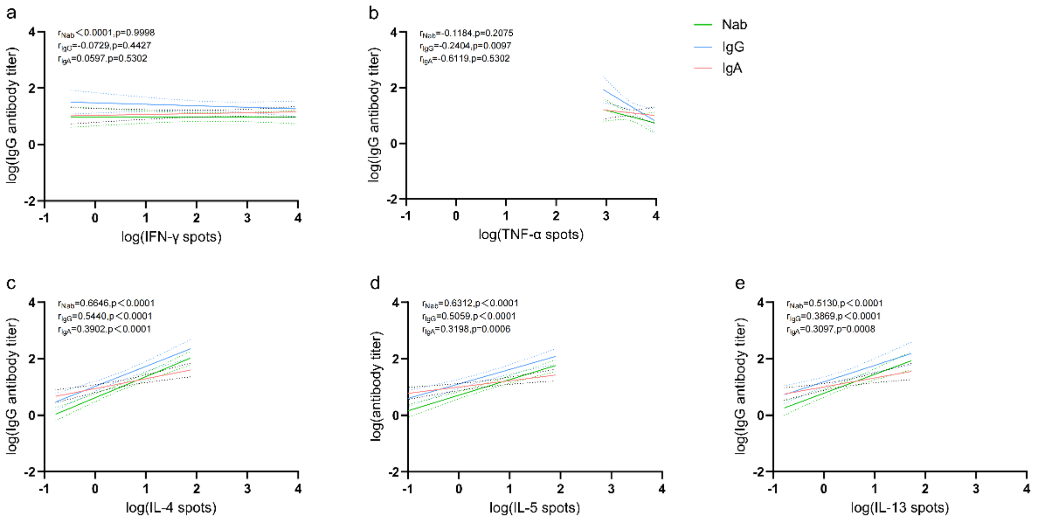

The correlation between IFN-γ(a), TNF-α(b), IL-4(c), IL-5(d) and IL-13(e) producing cells and antibodies.

Table S1 Severe adverse events after vaccination.

Group	Sex	Age	Symptom	Type of serious adverse events	Outcome	Related to vaccines
Phase 1 trial						
MD	Female	67	Death for car accident	Death	Death	No
SD	Female	34	Plasma cell mastitis	Hospitalization or prolonged hospitalization	Symptom persisting	No
HD	Female	64	Lumbar fracture	Hospitalization or prolonged hospitalization	Symptom resolved	No
HD	Female	64	Myelodysplastic syndrome	Hospitalization or prolonged hospitalization	Symptom persisting	Yes
HD	Female	64	Atrophic gastritis	Hospitalization or prolonged hospitalization	Symptom persisting	No
MIX	Female	69	Infiltrating lung adenocarcinoma	Hospitalization or prolonged hospitalization	Symptom persisting	No
MIX	Male	53	Klebsiella pneumoniae pneumonia	Hospitalization or prolonged hospitalization	Symptom persisting	No

Group	Sex	Age	Symptom	Type of serious adverse events	Outcome	Related to vaccines
MIX	Male	53	Cerebral hemorrhage	Hospitalization or prolonged hospitalization	Symptom persisting	No
MIX	Male	53	Cerebral infarction	Hospitalization or prolonged hospitalization	Symptom persisting	No
MIX	Male	53	Hydrocephalus	Hospitalization or prolonged hospitalization	Symptom persisting	No
MIX	Male	53	Hydronephrosis	Hospitalization or prolonged hospitalization	Symptom persisting	No
MIX	Male	53	Nephrolithiasis	Hospitalization or prolonged hospitalization	Symptom persisting	No
MIX	Male	73	Cerebral infarction	Hospitalization or prolonged hospitalization	Symptom persisting	No
Placebo	Female	62	Comminuted fracture	Hospitalization or prolonged hospitalization	Symptom persisting	No
Placebo	Female	52	Rheumatoid arthritis	Hospitalization or prolonged hospitalization	Symptom persisting	No
Phase 2 trial						
MD	Male	51	Atrophy of eyeball	Hospitalization or prolonged hospitalization	Symptom resolved	No
MD	Male	54	Tendon rupture	Hospitalization or prolonged hospitalization	Symptom resolved	No
MD	Female	63	Cholangioepithelial cell carcinoma	Hospitalization or prolonged hospitalization	Symptom persisting	No
MD	Female	63	Ileus	Hospitalization or prolonged hospitalization	Symptom persisting	No
HD	Female	78	Cerebral hemorrhage	Death	Death	No
HD	Female	63	Cerebral infarction	Hospitalization or prolonged hospitalization	Symptom persisting	No
HD	Female	64	Cataract	Hospitalization or prolonged hospitalization	Symptom resolved	No
HD	Female	64	Cataract	Hospitalization or prolonged hospitalization	Symptom resolved	No
HD	Female	66	Vertebrobasilar insufficiency	Hospitalization or prolonged hospitalization	Symptom persisting	No
HD	Female	66	Coronary stenosis	Hospitalization or prolonged hospitalization	Symptom persisting	No
HD	Female	66	Cardiac failure	Hospitalization or prolonged hospitalization	Symptom persisting	No
HD	Female	77	Acute myocardial infarction	Death	Death	No
HD	Female	70	Cerebral infarction	Hospitalization or prolonged hospitalization	Symptom persisting	No
MIX	Male	61	Craniocerebral injury	Hospitalization or prolonged hospitalization	Symptom resolved	No
MIX	Male	61	Cerebral concussion	Hospitalization or prolonged hospitalization	Symptom resolved	No
MIX	Male	61	Soft tissue injury	Hospitalization or prolonged hospitalization	Symptom resolved	No
IM	Female	34	Bruise	Hospitalization or prolonged hospitalization	Symptom resolved	No
IM	Female	34	Soft tissue contusion	Hospitalization or prolonged hospitalization	Symptom resolved	No
SD	Female	35	Tendon rupture	Hospitalization or prolonged hospitalization	Symptom persisting	No
SD	Female	35	Georgegumann	Hospitalization or prolonged hospitalization	Symptom persisting	No
SD	Male	45	Urinary tract infection	Hospitalization or prolonged hospitalization	Symptom resolved	No
SD	Male	75	Infectious pneumonia	Hospitalization or prolonged hospitalization	Symptom persisting	No
SD	Male	75	Chronic obstructive pulmonary disease	Hospitalization or prolonged hospitalization	Symptom persisting	No

Group	Sex	Age	Symptom	Type of serious adverse events	Outcome	Related to vaccines
SD	Male	75	Hydrothorax	Hospitalization or prolonged hospitalization	Symptom persisting	No
SD	Male	75	Congestive heart failure	Hospitalization or prolonged hospitalization	Symptom persisting	No
SD	Male	75	Coronary sclerosis	Hospitalization or prolonged hospitalization	Symptom persisting	No
SD	Male	75	Stenocardia	Hospitalization or prolonged hospitalization	Symptom persisting	No
SD	Male	75	Cardiac failure	Hospitalization or prolonged hospitalization	Symptom persisting	No
SD	Male	64	Radial fracture	Hospitalization or prolonged hospitalization	Symptom resolved	No
SD	Male	65	Acute myocardial infarction	Death	Death	No

Table S2 Adverse reactions within 14 days after vaccination in phase 1 trial.

	Low dose (N=18)	Middle dose (N=18)	High dose (N=18)	Mixed (N=18)	Single dose (N=17)	Placebo (N=30)	P value
Any adverse reactions							
Total	2(11.11)	4(22.22)	7(38.89)	5(27.78)	1(5.88)	5(16.67)	0.183
Grade≥3	0	0	0	1(5.56)	0	0	0.748
Solicited adverse reactions							
Total	0	4(22.22)	6(33.33)	5(27.78)	1(5.88)	5(16.67)	**0.048**
Grade≥3	0	0	0	1(5.56)	0	0	0.748
Local adverse reactions							
Total	0	0	1(5.56)	2(11.11)	1(5.88)	1(3.33)	0.676
Dysphonia	0	0	1(5.56)	0	1(5.88)	1(3.33)	0.787
Pain	0	0	0	2(11.11)	0	0	0.107
Dry mouth	0	0	0	0	0	1(3.33)	>0.999
Systematic adverse reactions							
Total	0	4(22.22)	6(33.33)	3(16.67)	0	5(16.67)	**0.022**
Grade≥3	0	0	0	1(5.56)	0	0	0.748
Fatigue	0	2(11.11)	4(22.22)	1(5.56)	0	0	**0.013**
Fever	0	2(11.11)	2(11.11)	1(5.56)	0	0	0.155
Grade≥3	0	0	0	1(5.56)	0	0	0.748
Diarrhea	0	0	1(5.56)	1(5.56)	0	4(13.33)	0.317
Joint pain	0	0	1(5.56)	1(5.56)	0	0	0.558
Cough	0	1(5.56)	1(5.56)	0	0	0	0.558
Sneeze	0	1(5.56)	1(5.56)	0	0	0	0.558
Headache	0	1(5.56)	1(5.56)	0	0	0	0.558
Pruritus	0	1(5.56)	0	1(5.56)	0	0	0.558
Runny nose	0	1(5.56)	0	0	0	0	0.748
Vomit	0	1(5.56)	0	0	0	0	0.748
Decreased appetite	0	1(5.56)	0	0	0	1(3.33)	>0.999
Pharyngalgia	0	0	0	0	0	1(3.33)	>0.999
Unsolicited adverse reactions							
Total	2(11.11)	0	0	0	0	0	0.107

Table S3 Adverse reactions within 14 days after vaccination in phase 2 trial.

	18-59 years old								⩾60 years old							
	B1 (N=50)	B2 (N=48)	B3 (N=50)	B4 (N=50)	B5 (N=50)	B6 (N=50)	Placebo 1(N=60)	P value	B7 (N=49)	B8 (N=51)	B9 (N=50)	B10 (N=49)	B11 (N=50)	B12 (N=50)	Placebo 2(N=60)	P value
Any adverse reactions																
Total	18(36.00)	15(31.25)	12(24.00)	23(46.00)	25(50.00)	9(18.00)	14(23.33)	**0.003**	7(14.29)	2(3.92)	3(6.00)	1(2.04)	7(14.00)	2(4.00)	5(8.33)	0.108
Grade≥3	1(2.00)	1(2.08)	4(8.00)	0	0	0	0	**0.015**								
Solicited																
Total	17(34.00)	14(29.17)	12(24.00)	23(46.00)	25(50.00)	9(18.00)	13(21.67)	**0.002**	6(12.24)	2(3.92)	3(6.00)	0	7(14.00)	2(4.00)	5(8.33)	0.081
Grade≥3	1(2.00)	1(2.08)	4(8.00)	0	0	0	0	**0.015**								
Local adverse reactions																
Total	5(10.00)	7(14.58)	5(10.00)	15(30.00)	19(38.00)	5(10.00)	5(8.33)	**<0.001**	0	0	1(2.00)	0	3(6.00)	1(2.00)	2(3.33)	0.249
Pain	0	0	0	15(30.00)	18(36.00)	0	1(1.67)	**<0.001**	0	0	0	0	2(4.00)	0	0	0.053
Dry mouth	4(8.00)	4(8.33)	5(10.00)	0	0	5(10.00)	4(6.67)	0.131	0	0	1(2.00)	0	0	1(2.00)	1(1.67)	0.702
Pharyngeal swelling	3(6.00)	2(4.17)	1(2.00)	0	0	0	3(5.00)	0.207								
Swelling	0	0	0	0	3(6.00)	0	0	**0.005**								
Pruritus	0	0	0	1(2.00)	2(4.00)	0	0	0.173	0	0	0	0	1(2.00)	0	0	0.401
Dysphonia	1(2.00)	0	1(2.00)	0	0	0	0	0.520	0	0	0	0	0	0	1(1.67)	0.544
Induration	0	0	0	1(2.00)	1(2.00)	0	0	0.520								
Oral mucositis	0	1(2.08)	0	0	0	0	1(1.67)	0.578	0	0	0	0	0	0	1(1.67)	0.544
Redness	0	0	0	1(2.00)	0	0	0	0.404								
Systematic adverse reactions																
Total	17(34.00)	12(25.00)	11(22.00)	14(28.00)	14(28.00)	5(10.00)	11(18.33)	0.113	6(12.24)	2(3.92)	3(6.00)	0	5(10.00)	2(4.00)	5(8.33)	0.197
Grade≥3	1(2.00)	1(2.08)	4(8.00)	0	0	0	0	**0.015**								
Fatigue	4(8.00)	5(10.42)	7(14.00)	5(10.00)	9(18.00)	1(2.00)	5(8.33)	0.212	3(6.12)	0	2(4.00)	0	1(2.00)	2(4.00)	3(5.00)	0.429
Fever	4(8.00)	6(12.50)	7(14.00)	3(6.00)	4(8.00)	2(4.00)	1(1.67)	0.188	1(2.04)	2(3.92)	0	0	2(4.00)	0	1(1.67)	0.447
Grade≥3	1(2.00)	1(2.08)	4(8.00)	0	0	0	0	**0.015**								
Headache	6(12.00)	4(8.33)	2(4.00)	4(8.00)	3(6.00)	1(2.00)	2(3.33)	0.382	1(2.04)	0	1(2.00)	0	0	0	0	0.508
Diarrhea	5(10.00)	2(4.17)	0	3(6.00)	0	1(2.00)	1(1.67)	0.056	2(4.08)	0	1(2.00)	0	1(2.00)	0	1(1.67)	0.544
Joint pain	4(8.00)	2(4.17)	2(4.00)	0	3(6.00)	0	3(5.00)	0.314	1(2.04)	0	1(2.00)	0	1(2.00)	0	1(1.67)	0.815
Oropharyngeal pain	3(6.00)	3(6.25)	3(6.00)	2(4.00)	2(4.00)	0	4(6.67)	0.719								
Myalgia	2(4.00)	1(2.08)	1(2.00)	3(6.00)	3(6.00)	0	0	0.284	1(2.04)	0	0	0	0	0	1(1.67)	0.585

Nausea	2(4.00)	2(4.17)	0	0	2(4.00)	0	2(3.33)	0.422	3(6.12)	0	0	0	0	0	0	**0.004**
Cough	2(4.00)	1(2.08)	1(2.00)	2(4.00)	1(2.00)	0	1(1.67)	0.847	0	0	2(4.00)	0	0	0	1(1.67)	0.195
Runny nose	1(2.00)	2(4.17)	2(4.00)	1(2.00)	1(1.00)	0	0	0.397	0	0	0	0	1(2.00)	0	0	0.401
Sneeze	3(6.00)	2(4.17)	1(2.00)	1(2.00)	0	0	0	0.192								
Decreased appetite	1(2.00)	2(4.17)	0	0	0	1(2.00)	0	0.310	0	0	0	0	0	1(2.00)	1(1.67)	0.595
Pruritus	1(2.00)	0	1(2.00)	3(6.00)	0	0	1(1.67)	0.220								
Chest pain	2(4.00)	0	0	1(2.00)	0	0	1(1.67)	0.368	1(2.04)	0	0	0	0	0	0	0.386
Vomit	0	2(4.17)	0	0	0	0	1(1.67)	0.173	1(2.04)	0	0	0	0	0	0	0.386
Unsolicited adverse reactions																
Total	3(6.00)	2(4.17)	1(2.00)	0	3(6.00)	1(2.00)	2(3.33)	0.601	2(4.08)	0	0	1(2.04)	0	0	0	0.158

Table S4 Unsolicited adverse reactions in phase 2 trial

	Low dose (N=18)	Middle dose (N=18)	High dose (N=18)	Mixed (N=18)	Single dose (N=17)	Placebo (N=30)	P value
phase 1 trial							
Total	2(11.11)	0	0	0	0	0	0.107
Dizzy	2(11.11)	0	0	0	0	0	0.107

	Low dose	Middle dose	High dose	Mixed	Intramuscular	Single dose	Placebo	P value
18-59 years old								
N	50	48	50	50	50	50	60	
Total	3(6.00)	2(4.17)	1(2.00)	0	3(6.00)	1(2.00)	2(3.33)	0.601
Dizzy	1(2.00)	1(2.08)	0	1(1.01)	0	0	1(1.67)	0.691
Palpitation	1(2.00)	0	0	0	1(2.00)	0	0	0.520
Hidrosis	0	0	0	0	1(2.00)	0	0	0.404
Myospasm	0	0	0	0	1(2.00)	0	0	0.404
Myalgia	0	0	0	0	1(2.00)	0	0	0.404
Insomnia	1(2.00)	0	0	0	0	0	0	0.404
Chest discomfort	0	0	0	0	0	1(2.00)	0	0.404
Swirl	0	0	1(2.00)	0	0	0	0	0.404
Toothache	0	1(2.08)	0	0	0	0	0	0.372
Dysphagia	0	0	0	0	0	0	1(1.67)	0.546
≥60 years old								
N	49	51	50	49	50	50	60	
Total	2(4.08)	0	0	1(2.04)	0	0	0	0.158
Dizzy	1(2.04)	0	0	1(2.04)	0	0	0	0.499
Dyspnea	1(1.01)	0	0	0	0	0	0	0.396

Table S5. GMT, GMFI and Seroconversion rates of neutralising antibodies to live SARS-CoV-2 in the phase 1 trial.

	Low dose (N=18)	Middle dose (N=18)	High dose (N=18)	Mixed (N=18)	Single dose (N=17)	Placebo (N=30)	P value
Day 0 before 1st dose							
GMT	2.00	2.21 (1.91,2.56)	2.00	2.00	2.00	2.00	0.0525
Day 28 post 1st dose							
GMT					5.06 (2.68,9.53)	2.00	0.0837
GMFI					2.53 (1.34,4.77)	1.00	0.0837
Seroconversion rate (n, %)					8,47.06% (26.17,69.04)	0 (0.00,39.03)	0.0582
Day 28 post 2nd dose							
GMT	7.93 (4.14,15.19)	14.36 (4.84,42.61)	26.32 (10.57,65.54)	57.03 (23.95,135.80)		2.12 (1.88,2.41)	**<0.0001**
GMFI	3.96 (2.07,7.59)	6.46 (2.38,17.53)	13.16 (5.28,32.77)	28.52 (11.98,67.90)		1.06 (0.94,1.20)	**<0.0001**
Seroconversion rate (n, %)	12,66.67% (43.75,83.72)	10,58.82% (36.01,78.39)	15,83.34% (60.78,94.16)	15,93.75% (71.67,98.89)		1,4.35% (0.77,20.99)	**<0.0001**

There were no endpoints related to the second dose in single dose group.

Table S6 GMT, GMFI and Seroconversion rates of SARS-CoV-2 RBD-specific IgG in the phase 1 trial.

	Low dose (N=18)	Middle dose (N=18)	High dose (N=18)	Mixed (N=18)	Single dose (N=17)	Placebo (N=30)	P value
Day 0 before 1st dose							
GMT	5.00	5.00	5.00	5.00	5.00	5.36 (4.65,6.18)	0.7131
Day 28 post 1st dose							

GMT	6.80 (4.43,10.46)	6.80 (4.58,10.11)	12.60 (6.67,23.82)	71.27 (35.68,142.37)	6.93 (4.84,9.92)	5.24 (4.76,5.76)	**<0.0001**
GMFI	1.36 (0.89,2.09)	1.36 (0.92,2.02)	2.52 (1.33,4.76)	14.25 (7.14,28.47)	1.39 (0.97,1.98)	0.98 (0.93,1.02)	**<0.0001**
Seroconversion rate (n, %)	3,16.67% (5.84,39.22)	3,16.67% (5.84,39.22)	9,50.00% (29.03,70.97)	17,94.44% (74.24,99.01)	5,29.41% (13.28,53.13)	0 (0.00,11.35)	**<0.0001**
Day 0 before 2nd dose							
GMT	6.80 (4.17,11.11)	5.83 (4.53,7.51)	9.62 (5.56,16.64)	35.13 (17.38,70.99)		5.15 (4.84,5.49)	**<0.0001**
GMFI	1.36 (0.83,2.22)	1.17 (0.91,1.50)	1.92 (1.11,3.33)	7.025 (3.48,14.20)		0.94 (0.83,1.07)	**<0.0001**
Seroconversion rate (n, %)	3,16.67% (5.84,39.22)	2,11.11% (3.10,32.80)	8,44.44% (24.56,66.28)	14,87.50% (63.98,96.50)		0 (0.00,14.31)	**<0.0001**
Day 14 post 2nd dose							
GMT	25.20 (8.99,70.63)	18.52 (6.31,54.33)	76.98 (26.14,226.72)	128.84 (47.95,346.21)		5.15 (4.84,5.49)	**<0.0001**
GMFI	5.04 (1.80,14.13)	3.70 (1.26,10.87)	15.40 (5.23,45.34)	25.77 (9.59,69.24)		0.94 (0.83,1.07)	**<0.0001**
Seroconversion rate (n, %)	9,50.00% (29.03,70.97)	6,33.33% (16.28,56.25)	13,72.22% (49.13,87.50)	14,87.50% (63.98,96.50)		0 (0.00,14.31)	**<0.0001**
Day 28 post 2nd dose							
GMT	25.20 (8.99,70.63)	15.03 (5.28,42.84)	76.98 (26.48,223.78)	320.00 (136.37,750.92)		5.31 (4.69,6.02)	**<0.0001**
GMFI	5.04 (1.80,14.13)	3.01 (1.06,8.57)	15.40 (5.30,44.76)	64.00 (27.27,150.19)		0.97 (0.91,1.03)	**<0.0001**
Seroconversion rate (n, %)	9,50.00% (29.03,70.97)	5,29.41% (13.28,53.13)	13,72.22% (49.13,87.50)	15,93.75% (71.67,98.89)		0 (0.00,14.31)	**<0.0001**

There were no endpoints related to the second dose in single dose group.

Table S7 GMT, GMFI and Seroconversion rates of sera IgA in the phase 1 trial.

	Low dose (N=18)	Middle dose	High dose (N=18)	Mixed (N=18)	Single dose	Placebo (N=30)	P value

		(N=18)			(N=17)		
Day 0 before 1st dose							
GMT	5.61 (4.92,6.41)	5.40 (4.83,6.04)	8.91 (4.98,15.95)	5.83 (4.83,7.05)	5.89 (4.82,7.20)	7.58 (5.53,10.39)	0.1360
Day 28 post 1st dose							
GMT	6.30 (4.97,7.98)	7.35 (5.13,10.52)	20.79 (10.82,39.92)	26.19 (15.83,43.33)	7.83 (5.37,11.41)	7.41 (5.44,10.09)	**<0.0001**
GMFI	1.12 (0.86,1.47)	1.36 (0.97,1.91)	2.33 (1.30,4.19)	4.49 (2.64,7.64)	1.33 (0.95,1.86)	0.98 (0.88,1.09)	**<0.0001**
Seroconversion rate (n, %)	3,16.67% (5.84,39.22)	4,22.22% (9.00,45.21)	8,44.44% (24.56,66.28)	13,72.22% (49.13,87.50)	4,23.53% (9.56,47.26)	1,3.33% (0.59,16.67)	**<0.0001**
Day 0 before 2nd dose							
GMT	6.80 (5.07,9.14)	8.91 (5.23,15.17)	21.60 (8.93,52.28)	17.56 (11.18,27.59)		7.86 (5.44,11.36)	**0.0050**
GMFI	1.21 (0.89,1.65)	1.65 (1.02,2.68)	2.43 (1.11,5.30)	2.95 (1.85,4.71)		0.94 (0.81,1.10)	**0.0017**
Seroconversion rate (n, %)	3,16.67% (5.84,39.22)	5,27.78% (12.50,50.87)	5,27.78% (12.50,50.87)	13,81.25% (56.99,93.41)		1,4.35% (0.77,20.99)	**<0.0001**
Day 14 post 2nd dose							
GMT	8.91 (5.38,14.76)	11.67 (5.55,24.54)	44.90 (16.10,125.21)	25.94 (14.67,45.87)		8.35 (5.62,12.40)	**0.0006**
GMFI	1.59 (0.94,2.69)	2.16 (1.09,4.30)	5.04 (1.96,12.98)	4.36 (2.47,7.72)		1.00 (0.86,1.17)	**0.0003**
Seroconversion rate (n, %)	6,33.33% (16.28,56.25)	7,38.89% (20.31,61.38)	11,61.11% (38.62,79.69)	12,75.00% (50.50,89.82)		2,8.70% (2.42,26.80)	**0.0003**
Day 28 post 2nd dose							
GMT	9.26 (5.47,15.69)	13.30 (6.12,28.94)	30.55 (10.07,92.69)	25.94 (13.06,51.51)		8.10 (5.23,12.54)	**0.0189**
GMFI	1.65 (0.95,2.87)	2.45 (1.19,5.04)	3.43 (1.24,9.45)	4.36 (2.17,8.78)		0.97 (0.84,1.12)	**0.0048**
Seroconversion rate (n, %)	6,33.33% (16.28,56.25)	8,47.06% (26.17,69.04)	5,27.78% (12.50,50.87)	10,62.50% (38.64,81.52)		0.00 (0.00,14.31)	**0.0006**

There were no endpoints related to the second dose in single dose groups.

Table S8 GMT, GMFI and Seroconversion rates of neutralising antibodies to live SARS-CoV-2 in the phase 2 trial.

	Low dose	Middle dose	High dose	Mixed	Intramuscular	Single dose	Placebo	P value
Total								
N	98	95	99	99	100	100	120	
Day 0 before 1st dose								
GMT	2.04 (1.96,2.12)	2.00	2.00	2.00	2.00	2.00	2.00	0.3719
Day 28 post 1st dose								
GMT					3.33 (2.91,3.82)	2.54 (2.28,2.83)	2.27 (1.98,2.60)	**0.0004**
GMFI					1.66 (1.45,1.90)	1.27 (1.14,1.41)	1.14 (0.99,1.30)	**<0.0001**
Seroconversion rate (n, %)					42,42.00% (32.80,51.79)	20,20.41% (13.62,29.43)	4,10.26% (4.06,23.58)	**<0.0001**
Day 28 post 2nd dose								
GMT	5.99 (4.33,8.30)	6.15 (4.56,8.30)	21.77 (15.13,31.32)	97.37 (74.30,127.59)			2.36 (2.06,2.71)	**<0.0001**
GMFI	3.01 (2.18,4.154)	3.08 (2.28,4.15)	10.88 (7.56,15.66)	48.68 (37.15,63.80)			1.18 (1.03,1.35)	**<0.0001**
Seroconversion rate (n, %)	40,43.48% (33.81,53.67)	47,51.09% (41.04,61.05)	78,79.59% (70.57,86.38)	95,97.94% (92.79,99.43)			9,11.54% (6.19,20.50)	**<0.0001**
18-59 years old								
N	50	47	50	50	50	50	60	
Day 0 before 1st dose								
GMT	2.07 (1.93,2.23)	2.00	2.00	2.00	2.00	2.00	2.00	0.4251
Day 28 post 1st dose								
GMT					3.47 (2.85,4.22)	2.06 (1.98,2.14)	2.00	**<0.0001**
GMFI					1.73 (1.42,2.11)	1.03 (0.99,1.07)	1.00	**<0.0001**
Seroconversion rate (n, %)					22,44.00% (31.16,57.69)	2,4.00% (1.10,13.46)	0 (0.00,16.82)	**<0.0001**
Day 28 post 2nd dose								
GMT	6.52	6.21	25.36	124.97			2.41	**<0.0001**

	(4.04,10.51)	(4.02,9.58)	(15.16,42.45)	(85.45,182.76)			(1.89,3.07)	
GMFI	3.14 (1.99,4.96)	3.10 (2.01,4.79)	12.68 (7.58,21.22)	62.48 (42.72,91.38)			1.20 (0.94,1.54)	**<0.0001**
Seroconversion rate (n, %)	23,46.94% (33.70,60.62)	25,55.56% (41.18,69.06)	42,85.71% (73.33,92.90)	47,97.92% (89.10,99.63)			3,7.89% (2.72,20.80)	**<0.0001**
≥60 years old								
N	48	48	49	49	50	50	60	
Day 0 before 1st dose								
GMT	2.00	2.00	2.00	2.00	2.00	2.00	2.00	
Day 28 post 1st dose								
GMT					3.19 (2.64,3.87)	3.16 (2.59,3.86)	2.56 (1.97,3.35)	0.4182
GMFI					1.60 (1.32,1.93)	1.58 (1.30,1.93)	1.28 (0.98,1.67)	0.4182
Seroconversion rate (n, %)					20,40.00% (27.61,53.82)	18,37.50% (25.22,51.64)	4,20.00% (8.07,41.60)	0.2697
Day 28 post 2nd dose								
GMT	5.47 (3.47,8.62)	6.11 (3.98,9.36)	18.68 (11.00,31.71)	76.25 (51.88,112.07)			2.32 (2.01,2.68)	**<0.0001**
GMFI	2.87 (1.79,4.59)	3.05 (1.99,4.68)	9.34 (5.50,15.85)	38.12 (25.94,56.03)			1.16 (1.01,1.34)	**<0.0001**
Seroconversion rate (n, %)	17,39.53% (26.37,54.42)	22,46.81% (33.33,60.77)	36,73.47% (59.74,83.79)	48,97.96% (89.31,99.64)			6,15.00% (7.06,29.07)	**<0.0001**

Table S9 GMT, GMFI and Seroconversion rates of SARS-CoV-2 RBD-specific IgG in the phase 2 trial.

	Low dose	Middle dose	High dose	Mixed	Intramuscular	Single dose	Placebo	P value
Total								
N	98	95	99	99	100	100	120	
Day 0 before 1st dose								
GMT	5.04 (4.97,5.11)	5.07 (4.93,5.22)	5.14 (4.92,5.37)	5.07 (4.97,5.17)	5.04 (4.97,5.11)	5.00	5.24 (4.94,5.55)	0.4735
Day 28 post 1st dose								
GMT					57.76 (43.79,76.17)	6.87 (5.97,7.92)	5.66 (4.40,7.28)	**<0.0001**

GMFI					11.47 (8.70,15.12)	1.38 (1.19,1.58)	1.13 (0.88,1.46)	**<0.0001**
Seroconversion rate (n, %)					91,91.00% (83.77,95.19)	22,22.45% (15.32,31.66)	1,2.56% (0.45,13.18)	**<0.0001**
Day 0 before 2nd dose								
GMT	6.27 (5.44,7.23)	5.99 (5.23,6.86)	6.78 (5.86,7.84)	40.86 (30.46,54.80)			5.42 (4.96,5.91)	**<0.0001**
GMFI	1.25 (1.09,1.42)	1.18 (1.03,1.35)	1.32 (1.13,1.53)	8.06 (6.02,10.78)			1.01 (0.97,1.05)	**<0.0001**
Seroconversion rate (n, %)	12,12.63% (11.48,33.64)	8,8.70% (4.47,16.23)	20,20.41% (13.62,29.43)	85,86.73% (78.62,92.08)			1,1.28% (0.23,6.91)	**<0.0001**
Day 28 post 2nd dose								
GMT	22.15 (14.95,32.83)	23.43 (15.64,35.11)	42.03 (27.28,64.76)	326.93 (246.09,434.34)			5.37 (4.95,5.83)	**<0.0001**
GMFI	4.40 (2.97,6.50)	4.62 (3.07,6.93)	8.17 (5.28,12.64)	64.46 (48.59,85.51)			1.00 (0.97,1.03)	**<0.0001**
Seroconversion rate (n, %)	44,46.32% (36.63,56.29)	47,51.09% (41.04,61.05)	59,60.20% (50.30,69.33)	95,97.94% (92.79,99.43)			1,1.28% (0.23,6.91)	**<0.0001**
18-59 years old								
N	50	47	50	50	50	50	60	
Day 0 before 1st dose								
GMT	5.07 (4.93,5.21)	5.15 (4.83,5.47)	5.07 (4.93,5.21)	5.07 (4.93,5.21)	5.07 (4.93,5.21)	5.00	5.24 (4.77,5.74)	0.8945
Day 28 post 1st dose								
GMT					85.74 (60.57,121.37)	6.60 (5.474,7.95)	5.00	**<0.0001**
GMFI					16.91 (11.96,23.92)	1.32 (1.10,1.59)	1.00	**<0.0001**
Seroconversion rate (n, %)					48,96.00% (86.54,98.90)	9,18.00% (9.77,30.80)	0 (0.00,16.82)	**<0.0001**
Day 0 before 2nd dose								
GMT	7.22 (5.64,9.25)	6.60 (5.10,8.53)	7.02 (5.68,8.68)	40.00 (27.20,58.82)			5.38 (4.64,6.24)	**<0.0001**
GMFI	1.42 (1.13,1.79)	1.27 (0.99,1.66)	1.39 (1.12,1.72)	7.89 (5.35,11.64)			1.00	**<0.0001**
Seroconversion rate (n, %)	10,20.41% (11.48,33.64)	5,11.11% (4.84,23.50)	13,26.53% (16.21,40.26)	44,89.80% (78.24,95.56)			0 (0.00,9.18)	**<0.0001**
Day 28 post 2nd dose								
GMT	27.30 (14.90,50.03)	21.27 (12.20,37.09)	56.97 (30.55,106.24)	472.58 (320.91,695.94)			5.48 (4.71,6.37)	**<0.0001**

GMFI	5.38 (2.95,9.81)	4.13 (2.35,7.25)	11.23 (6.01,21.01)	93.16 (63.26,137.21)			1.02 (0.98,1.06)	**<0.0001**
Seroconversion rate (n, %)	24,48.98% (35.58,62.53)	22,48.89% (34.96,63.00)	32,65.31% (51.31,77.08)	47,97.92% (89.10,99.63)			1,2.63% (0.47,13.49)	**<0.0001**
≥60 years old								
N	48	48	49	49	50	50	60	
Day 0 before 1st dose								
GMT	5.00	5.00	5.22 (4.79,5.68)	5.07 (4.93,5.22)	5.00	5.00	5.24 (4.87,5.63)	0.5255
Day 28 post 1st dose								
GMT					38.91 (25.73,58.83)	7.17 (5.76,8.94)	6.37 (3.84,10.59)	**<0.0001**
GMFI					7.78 (5.15,11.77)	1.44 (1.15,1.79)	1.28 (0.77,2.12)	**<0.0001**
Seroconversion rate (n, %)					43,86.00% (73.81,93.05)	13,27.08% (16.57,41.00)	1,5.00% (0.89,23.61)	**<0.0001**
Day 0 before 2nd dose								
GMT	5.39 (4.76,6.11)	5.46 (4.90,6.09)	6.54 (5.32,8.04)	41.73 (26.43,65.91)			5.45 (4.92,6.04)	**<0.0001**
GMFI	1.08 (0.95,1.22)	1.09 (0.98,1.22)	1.25 (1.01,1.56)	8.23 (5.25,12.89)			1.02 (0.94,1.10)	**<0.0001**
Seroconversion rate (n, %)	2,4.35% (1.20,14.53)	3,6.38% (2.19,17.16)	7,14.29% (7.10,26.67)	41,83.67% (70.96,91.49)			1,2.50% (0.44,12.88)	**<0.0001**
Day 28 post 2nd dose								
GMT	17.73 (10.64,29.53)	25.70 (14.03,47.07)	31.01 (16.86,57.02)	227.88 (152.36,340.83)			5.27 (4.87,5.69)	**<0.0001**
GMFI	3.55 (2.13,5.91)	5.14 (2.81,9.42)	5.94 (3.21,11.02)	44.94 (30.17,66.93)			0.98 (0.95,1.02)	**<0.0001**
Seroconversion rate (n, %)	20,43.48% (30.21,57.75)	25,53.19% (39.23,66.67)	27,55.10% (41.32,68.15)	48,97.96% (89.31,99.64)			0 (0.00,8.76)	**<0.0001**

Table S10 GMT, GMFI and Seroconversion rates of sera IgA in the phase 2 trial.

	Low dose	Middle dose	High dose	Mixed	Intramuscular	Single dose	Placebo	P value
Total								
N	98	95	99	99	100	100	120	

Day 0 before 1st dose								
GMT	5.80 (5.24,6.43)	5.91 (5.35,6.54)	5.96 (5.35,6.63)	6.43 (5.76,7.19)	6.16 (5.54,6.85)	5.99 (5.42,6.61)	6.02 (5.53,6.54)	0.8665
Day 28 post 1st dose								
GMT					20.71 (15.57,27.530)	9.79 (7.96,12.04)	5.87 (5.05,6.82)	**<0.0001**
GMFI					3.36 (2.57,4.40)	1.63 (1.37,1.94)	0.98 (0.86,1.12)	**<0.0001**
Seroconversion rate (n, %)					60,60.00% (44.23,70.62)	32,32.65% (17.47,41.67)	1,2.56% (0.45,13.18)	**<0.0001**
Day 0 before 2nd dose								
GMT	7.86 (6.47,9.54)	8.28 (6.89,9.96)	12.10 (9.38,15.63)	18.37 (13.80,24.45)			5.87 (5.32,6.47)	**<0.0001**
GMFI	1.35 (1.15,1.58)	1.39 (1.17,1.66)	2.03 (1.63,2.53)	2.85 (2.21,3.67)			0.98 (0.95,1.02)	**<0.0001**
Seroconversion rate (n, %)	20,21.05% (14.06,30.29)	19,20.65% (13.64,30.02)	36,36.73% (27.86,46.61)	46,46.94% (37.36,56.75)			0 (0.00,4.69)	**<0.0001**
Day 28 post 2nd dose								
GMT	13.99 (10.53,18.58)	10.86 (7.90,14.95)	13.65 (10.27,18.14)	22.91 (16.80,31.24)			5.32 (4.97,5.69)	**<0.0001**
GMFI	2.40 (1.84,3.14)	1.83 (1.32,2.54)	2.29 (1.73,3.02)	3.59 (2.63,4.91)			0.89 0.83,0.96)	**<0.0001**
Seroconversion rate (n, %)	42,44.21% (34.64,54.23)	20,21.74% (14.54,31.21)	40,40.82% (31.61,50.71)	56,57.73% (47.79,67.08)			1,1.28% (0.23,6.91)	**<0.0001**
18-59 years old								
N	50	47	50	50	50	50	60	
Day 0 before 1st dose								
GMT	5.36 (4.99,5.76)	5.80 (5.00,6.71)	5.99 (5.09,7.05)	6.07 (5.35,6.89)	6.33 (5.50,7.29)	6.33 (5.26,7.62)	5.95 (5.31,6.65)	0.6679
Day 28 post 1st dose								
GMT					21.44 (14.01,32.80)	10.57 (7.62,14.67)	5.38 (4.61,6.27)	**0.0001**
GMFI					3.39 (2.22,5.17)	1.67 (1.30,2.15)	0.93 (0.84,1.03)	**<0.0001**
Seroconversion rate (n, %)					29,58.00% (44.23,70.62)	14,28.00% (17.47,41.67)	0 (0.00,16.82)	**<0.0001**
Day 0 before 2nd dose								
GMT	8.32 (6.36,10.89)	9.55 (7.01,13.00)	16.41 (11.11,24.23)	15.07 (10.78,21.07)			5.79 (5.07,6.60)	**<0.0001**
GMFI	1.55	1.64	2.73	2.47			0.98	**<0.0001**

	(1.20,2.01)	(1.21,2.22)	(1.92,3.87)	(1.80,3.40)			(0.95,1.02)	
Seroconversion rate (n, %)	12,24.49% (14.60,38.09)	14,31.11% (19.53,45.66)	25,51.02% (37.47,64.42)	23,46.94% (33.70,60.62)			0 (0.00,9.18)	**<0.0001**
Day 28 post 2nd dose								
GMT	16.41 (10.76,25.03)	11.67 (7.22,18.85)	11.20 (7.82,16.05)	22.78 (14.99,34.62)			5.28 (4.87,5.73)	**<0.0001**
GMFI	3.06 (2.00,4.68)	2.00 (1.21,3.31)	1.86 (1.26,2.76)	3.83 (2.47,5.94)			0.90 (0.80,1.00)	**<0.0001**
Seroconversion rate (n, %)	26,53.06% (39.38,66.30)	11,24.44% (14.24,38.67)	17,34.69% (22.92,48.69)	29,60.42% (46.31,72.98)			0 (0.00,9.18)	**<0.0001**
≥60 years old								
N	48	48	49	49	50	50	60	
Day 0 before 1st dose								
GMT	6.30 (5.18,7.67)	6.03 (5.23,6.96)	5.93 (5.13,6.84)	6.83 (5.66,8.23)	5.99 (5.09,7.05)	5.66 (5.25,6.11)	6.09 (5.35,6.92)	0.7404
Day 28 post 1st dose								
GMT					20.00 (13.49,29.66)	9.04 (6.97,11.71)	6.37 (4.90,8.30)	**<0.0001**
GMFI					3.34 (2.36,4.73)	1.59 (1.23,2.05)	1.04 (0.81,1.32)	**<0.0001**
Seroconversion rate (n, %)					31,62.00% (48.15,74.14)	18,37.50% (25.22,51.64)	1,5.00% (0.89,23.61)	**<0.0001**
Day 0 before 2nd dose								
GMT	7.40 (5.54,9.89)	7.23 (5.85,8.93)	8.93 (6.48,12.31)	22.40 (14.01,35.81)			5.95 (5.13,6.90)	**<0.0001**
GMFI	1.16 (0.97,1.40)	1.19 (1.01,1.41)	1.51 (1.17,1.94)	3.28 (2.19,4.91)			0.98 (0.92,1.05)	**<0.0001**
Seroconversion rate (n, %)	8,17.39% (9.09,30.72)	5,10.64% (4.63,22.59)	11,22.45% (13.02,35.88)	23,46.94% (33.70,60.62)			0 (0.00,8.76)	**<0.0001**
Day 28 post 2nd dose								
GMT	11.80 (8.02,17.37)	10.15 (6.54,15.76)	16.64 (10.65,26.01)	23.04 (14.37,36.94)			5.36 (4.80,5.98)	**<0.0001**
GMFI	1.86 (1.35,2.55)	1.68 (1.08,2.61)	2.89 (1.88,4.19)	3.38 (2.14,5.33)			0.89 (0.79,0.99)	**<0.0001**
Seroconversion rate (n, %)	16,34.78% (22.68,49.23)	9,19.15% (10.42,32.54)	23,46.94% (33.70,60.62)	27,55.10% (41.32,68.15)			1,2.50% (0.44,12.88)	**<0.0001**

There were no endpoints related to the second dose in intramuscular and single dose group.
